# Toxicity Evaluation of Microemulsion (Nano Size) of Sour Cherry Kernel Extract for the Oral Bioavailability Enhancement

**DOI:** 10.17795/jjnpp-14370

**Published:** 2014-02-20

**Authors:** Anayatollah Salimi, Eisa Motaharitabar, Mehdi Goudarzi, Annahita Rezaie, Heibatullah Kalantari

**Affiliations:** 1 Department of Pharmaceutics, School of Pharmacy, Ahvaz Jundishapur University of Medical Sciences, Ahvaz, IR Iran; 2 Department of Pharmacology and Toxicology, School of Pharmacy, Ahvaz Jundishpur University of Medical Sciences, Ahvaz, IR Iran; 3 Department of pathobiology, School of Veterinary Medicine, Shahid Chamran University, Ahvaz, IR Iran

**Keywords:** Toxicity, Biological Availability, Biological Availability

## Abstract

**Background:**

In the recent years nanostructured materials have been the focus of researches due to their wide-spread possibilities to provide new shapes and structures for some materials. Microemulsions can provide uniform nano-sized droplets for templating. Microemulsions are isotropic, thermodynamically-stable systems of oil, water and surfactant with a 20-200 nm droplet size. They can be prepared as oil-in-water (o/w), water-in-oil (w/o) or bicontinuous systems, depending on the equilibrium spontaneous curvature of the surfactant layer at the oil-water interface.

**Objectives:**

The aim of this study was to introduce a system designed to improve and enhance the bioavailability of bioflavonoids in the *Prunus cerasus* (sour cherry) seed kernel extract by developing a novel delivery system, i.e. microemulsion (nanosized particles).

**Materials and Methods:**

Microemulsion formulations were prepared by mixing appropriate amounts of surfactants (Tween 80 and Span 20), cosurfactant (propylene glycol) (3:1 ratio), and oil phase (olive oil). The prepared microemulsions were evaluated regarding their mean droplet size, transparency, viscosity, and pH. Sour cherry kernel extract microemulsion was orally administered to mice at doses of 2.5%, 5%, and 10% for 10 days. On the last day, their blood as well as their liver and kidney were used for biochemical and histopathological analyses, respectively.

**Results:**

Biochemical factors levels and the pathological study indicated that there were not significant differences in microemulsion extracts compared with the control group (P > 0.05).

**Conclusions:**

Not only no toxicity evidence of this product was observed in the dose range used in foods or healthcare, but also it improved the cardiac function recovery.

## 1. Background

The effect of nanotechnology in medicine can mainly be seen in diagnostic methods, drug-release techniques, and regenerative medicine. In the recent years, nanostructured materials have been the focus of researches due to their wide-spread possibilities to provide new shapes and structures for some materials. Microemulsions are thermodynamically stable, low viscose mixtures of oil and water stabilized with a surfactant, and usually in combination with a cosurfactant. Microemulsions have shown several advantages for drug delivery such as; ease of preparation, increasing drug solubility, controlling drug delivery rate, perfect stability, improving bioavailability of hydrophilic and lipophilic drugs through different delivery routes. The mixture of water, oil, surfactant, and cosurfactant may lead to three types of microemulsions: water in oil, oil in water, and bicontinuous, with a wide variety of structures. Microemulsion droplet size ranges from 20 to 200 nm (1-6).

Perfect drug delivery properties and small droplet size of microemulsions are attributed to the solubility properties and their membranes penetration enhancement effects (5, 7-10). There is much interest in the function of compounds with plant sources, such as phytonutrients, which can increase the health quality. Development of a modern health system, medicinal plants, and natural products, plays an influential role in medicine (11-13). Extensive researches have been conducted on active ingredients for phytotherapy by many pharmaceutical companies for their potential medical values, such as sour cherry seed kernel extract in the cardioprotective mechanisms (14). Sour cherry plant is grown in Iran and many other countries. This fruit has nutritional consumption in Iran. Moreover, its stem and stone kernel are used for traditional healing of specific diseases. Sour cherry seed kernel extract is composed of two parts; 64-68% of the extract is a solid fraction, containing 2–4% cyanides, 1–3% polyphenols, 1–4% flavonoids, 1–3% vegetable acids, 1–2% pro and anthocyanidines, 1% stilbenes, 1% trans-resveratrol, and 1% catechins. 32-36% of the extract are oil fractions such as vegetable oils, including triglycerides, oleic acids, α-tocopherol, tocotrienols, and tocopherol-like components (15). Sour cherry seed kernel bioflavones are involved in some mechanisms including; (I) inducing the activity of cytoprotective enzyme heme oxygenase-1 (14, 15), (II) stimulating the production of cyclic guanosine-3,5-monophosphate (cGMP) (16, 17), (III) inducing the carbon monoxide (CO) production (15), (IV), reducing the caspase-3 activity (14). These mechanisms respectively cause: (I) severe reduction of oxidative tissue injury in many conditions (18, 19), (II) protection of cells from ischemic injury and improvement of cell survival (20), (III) prevention of cell death caused by mitochondrial calcium-overload (15), and (IV) other major tissue protective mechanisms (14). On the other hand, some fruit seeds contain receptacle poison compounds, for example, apricots contain compounds with potentiality of cyanide toxicity, and if consumed in adequate amounts, can reveal its effectiveness (21). Cyanide is found in abundance in *Prunus* species; the best characterized cyanogenic glycoside is perhaps amygdaline, which is present especially in the seeds and leaves of cherry, almond, peach, etc. These plants produce cyanogenic glycosides during their growing stage. For example, cherry kernel yields about 170 mg/100 g. Cyanogenic glycosides in plant tissues are toxic when they are decomposed by plant enzymes (Beta-glucosidases) or rumen microorganisms, and they can transform to toxic hydrocyanic acid, frequently called prussic acid and abbreviated as HCN (22). Cyanide can be readily bound to Fe, and inactivate cytochrome C oxidase (the last enzyme in the respiratory electron transport chain) in the mitochondrial membrane (23). In addition, flavonoids and polyphenolic compounds in large quantities can be toxic (24). The main purpose of this idea was to compose a microemulsion formulation at a nanoscale level to improve bioavailability of oral absorption of sour cherry seed kernel extract in mice. Sour cherry kernel could be improved through this delivery system.

## 2. Objectives

The goal of the present study was to test reactions of hepatotoxicity and nephrotoxicity in mice with orally-administrated (gavage) seed kernel extract microemulsion.

## 3. Materials and Methods

### 3.1. Chemicals

Span 20, Tween 80, and propylene glycol (PG) were obtained from Merck (Germany). Olive oil, ethanol, formalin, paraffin, and salt were purchased from a local market. All chemicals and solvents were of analytical grades.

### 3.2. Experimental Animals

A total of 50 Adult male albino mice (body weight: 25-30 g) were collected from the animal house of Ahvaz Jundishapur University of Medical Sciences, Iran. The investigation was performed under the local animal ethics committee guidelines of using experimental animals. Mice were 4-6 weeks old, kept in a conventional animal house. They were kept in a polycarbonate cage under the standard conditions (ambient temperature of 25 ± 2˚C) with approximately 12 hours of natural light per day. They were given ad libitum access to commercial food and water.

### 3.3. Sour Cherry Seed Extract Preparation

Sour cherry kernel seed extract was obtained from the pharmacology department of school of pharmacy of Debrecen Medical University. Seeds were dried, and their walls were removed. The kernel was crashed into small pieces, and extracted with n-hexane by a Soxhlet extractor for 4 hours. As a result, the oil fraction and solid fraction were separated. The solid kernel fraction was extracted with ethanol 70%, and the solvent was removed under vacuum using rotary evaporator at 40˚C temperature. Regarding the effective doses of sour cherry kernel extract in mice (250, 500 and 1000 mg/kg) (25), and the body weight of mice compared with the base microemulsion, we used the same doses. Therefore, we obtained 2.5%, 5%, and 10% extract microemulsions.

### 3.4. Microemulsions Preparation

**Figure 1.**
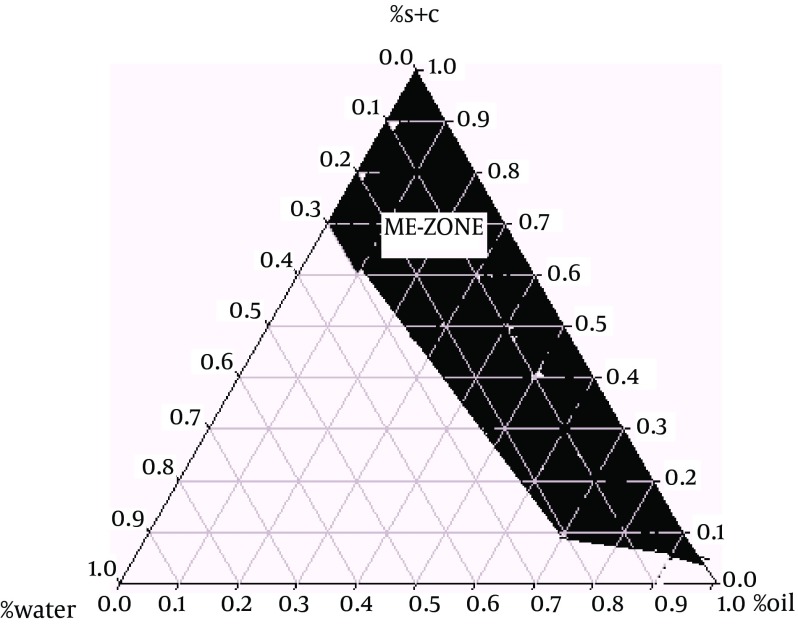
Pseudoternary Phase Diagram of Oil-Surfactant/Co-surfactant Mixture-Water System at the 3:1 Weight Ratio of Tween 80 Span 20/Propylene Glycol at Ambient Temperature, Dark Area Shows the Microemulsions Zone

Pseudoternary phase diagram was constructed by titration method to determine the components of the microemulsion. The system was composed of olive oil (oily phase), tween 80 and span 20 in the ratio of 1:1 as surfactants, and Propylene glycol as co-surfactant. The ratio of surfactant to co-surfactant was 3:1. In our experiment, we first used surfactant and co-surfactant in the ratio of 2:1, but after adding the crude extract to this system, transparency of the microemulsion was not clear enough. Therefore, we tried another system which consisted of surfactant/co-surfactant (3:1). A clear and transparent system was obtained after adding various doses of sour cherry kernel extract. Various microemulsions were selected from the pseudoternary phase diagram with 3:1 weight ratio, thus, 2.5%, 5%, and 10% doses of sour cherry kernel extract were produced. The stabilities of microemulsions were studied regarding the temperature and centrifugation. Finally, the microemulsion with the highest stability and better mean droplet size was selected and used in the experiments (Figure 1). The components of the suitable formulation are 35.42% oil, 60.66% surfactant-cosurfactant, and 3.901% water. Ratios of 2.5%, 5%, and 10% of the microemulsion formulations had the mean droplet sizes of 35.5 ± 1.1 nm, 6.32 ± 0.7, and 22.1 ± 0.09 nm, respectively. The microemulsion formulations had the average viscosity of 87.6 ± 3.5 centipoises, and the average pH of 5.6 ± 0.1.

#### 3.4.1. Viscosity Measurement

The viscosities of microemulsions were measured at 25˚C with a Brookfield viscometer (DV-II + Pro Brookfield, The USA) using spindle no. 34. with a shear rate of 100 rpm (26).

#### 3.4.2. Particle Size Measurement

The mean droplet size of microemulsions was measured by SCATTER SCOPE 1 QUIDIX (South Korea) at 25˚C, and their refractory indices were also computed (27).

#### 3.4.3. pH Determination

The pH values of the microemulsions were obtained by pH meter (Mettler Toledo SevenEasy, Switzerland) at 25˚C.

#### 3.4.4. Experimental Design

Mice were divided into five groups (10 mice per group), and received one unit of insulin per gram of their weight (in a 25 gram mouse, 25 units of insulin was administered) by oral gavage once daily for ten days. Group (I): (negative control), each mouse received only 0.2 mL of normal saline buffer; group (II**)**: the control group, each mouse received only base of microemulsion without extract; groups (III), (IV) and (V) received sour cherry kernel extract microemulsion in doses of 2.5%, 5%, and 10%, respectively.

#### 3.4.5. Sample Collection

Two hours after the last administration, mice were weighed, and anesthetized with diethyl ether, and then the mice were killed. Blood sample was withdrawn from the jugular vein and centrifuged. Afterwards, serum was separated; then the activities of serum enzymes such as aspartate aminotransferase (AST), alanine aminotransferase (ALT), and alkaline phosphatase (ALP) were measured to assess the liver function, and the levels of blood urea nitrogen (BUN) and creatinine were measured for kidney function evaluation. The enzyme activities were expressed in international units (IU), and for kidney factors in mg/dL. After bleeding, the abdomen was dissected; the liver and kidneys were removed, weighed, and fixed in 10% buffered formalin solution for histological studies.

### 3.5. Pathological Preparation

For light microscopy, tissues were removed, preprocessed by routine paraffin, sectioned (6 µm), and sections were stained with hematoxylin and eosin (H&E) to study the hepatotoxicity and nephrotoxicity. Staining was performed according to the standard instruction. Microscopic slides were evaluated by light microscope (Olympus BH2). Essential pictures were taken by MD130 camera, and photos were analyzed by quick photo micro 2.3 software.

#### 3.5.1. Toxicity Studies

The serum activities of ALT, AST, and ALP were assayed as indicators of liver function, according to the methods of Reitman, Frankel, and King, respectively (28, 29). BUN (blood urea nitrogen) and creatinine were measured as indicators of kidney function, according to the methods of Bretaudiere et al. and Spencer, respectively (30, 31).

### 3.6. Statistical Analysis

The collected data were analyzed using SPSS version 18.0. The results were expressed as Mean ± SD (standard deviation). One-way analysis of variance (ANOVA) followed by Tukey’s post-testing were applied to evaluate the significance of differences between the normal and sour cherry seed kernel extract microemulsion-received groups. P values < 0.05 were considered statistically significant.

## 4. Results

The results obtained in this study showed that animals in all groups did not have any physical appearance differences such as scabies, hair loss, soft or mucoid stool, and decreased defecation as compared with the control group. The results are shown in Tables 1 and 2. In general, there were no statistically significant differences in the enzyme activities of liver and kidney parameters (P ≥ 0.05). Regarding the initial and final body weight in the experiment process, there were not any significant differences. The liver and kidney weight did not show any differences in comparison with the values of the control group (P ≥ 0.05).

**Table 1.** Effect of Oral Administration of ME Extracts of Sour Cherry Kernel on the Biochemical Parameters ^a^ in Mice (Mean ± SD)

Groups	ALT, IU/L	AST, IU/L	ALP, IU/L	BUN, mg/dL	Cr, mg/dL
**Normal saline (I)**	65.9 ± 11.27	211.3 ± 15.81	175.9 ± 14.51	20.56 ± 2.79	0.24 ± 0.01
**Base ME (II)**	75.6 ± 11.39	219.6 ± 20.04	166.6 ± 13.67	19.35 ± 2.92	0.25 ± 0.01
2.5% extract ME (III)	66.9 ± 11.62	200.8 ± 18.62	179.8 ± 17.07	19.32 ± 2.66	0.25 ± 0.02
5% extract ME (IV)	71.4 ± 8.07	217 ± 19.28	176 ± 18.64	19.5 ± 2.14	0.24 ± 0.01
10% extract ME (V)	71.6 ± 10.14	208.1 ± 20.18	173.1 ± 17.49	19.35 ± 2.57	0.25 ± 0.02

^a^ Abbreviations: AST, aspartate aminotransferase; ALT, alanine aminotransferase; ALP, alkaline phosphatase; BUN, blood urea nitrogen; Cr, creatinine; ME, microemulsion.

All values are expressed as Mean ± SD for 10 mice in each group (P < 0.05 is the threshold for significant difference between the groups vs. negative control group). No significant difference was observed in any parameter.

**Table 2.** Effect of Oral Administration of ME Extracts of Sour Cherry Kernel on Body Weight (g), Liver, and a Single Kidney Weight (g) in Mice ^a^

Groups	Initial Body Weight, g	Final Body Weight, g	Liver Tissue Weight, g	Kidney Tissue Weight, g
**Normal saline (I)**	27.71 ± 1.29	29.12 ± 1.32	1.46 ± 0.18	0.31 ± 0.05
**Base ME (II)**	26.75 ± 1.15	28.27 ± 1.47	1.42 ± 0.20	0.30 ± 0.06
2.5% extract ME (III)	26.75 ± 1.86	28.05 ± 1.65	1.35 ± 0.19	0.29 ± 0.05
5% extract ME (IV)	28.45 ± 1.22	30.03 ± 1.09	1.51 ± 0.16	0.31 ± 0.07
10% extract ME (V)	27.82 ± 1.45	29.17 ± 1.53	1.35 ± 0.17	0.29 ± 0.06

^a^ Tested groups (II–V) versus negative control mice (no base ME and extract ME treatment). All values are expressed as Mean ± SD for 10 mice in each group (P value less than 0.05 is the threshold for significant difference between the groups with respect to each outcome variable). No significant difference was observed in any parameter.

**Figure 2.**
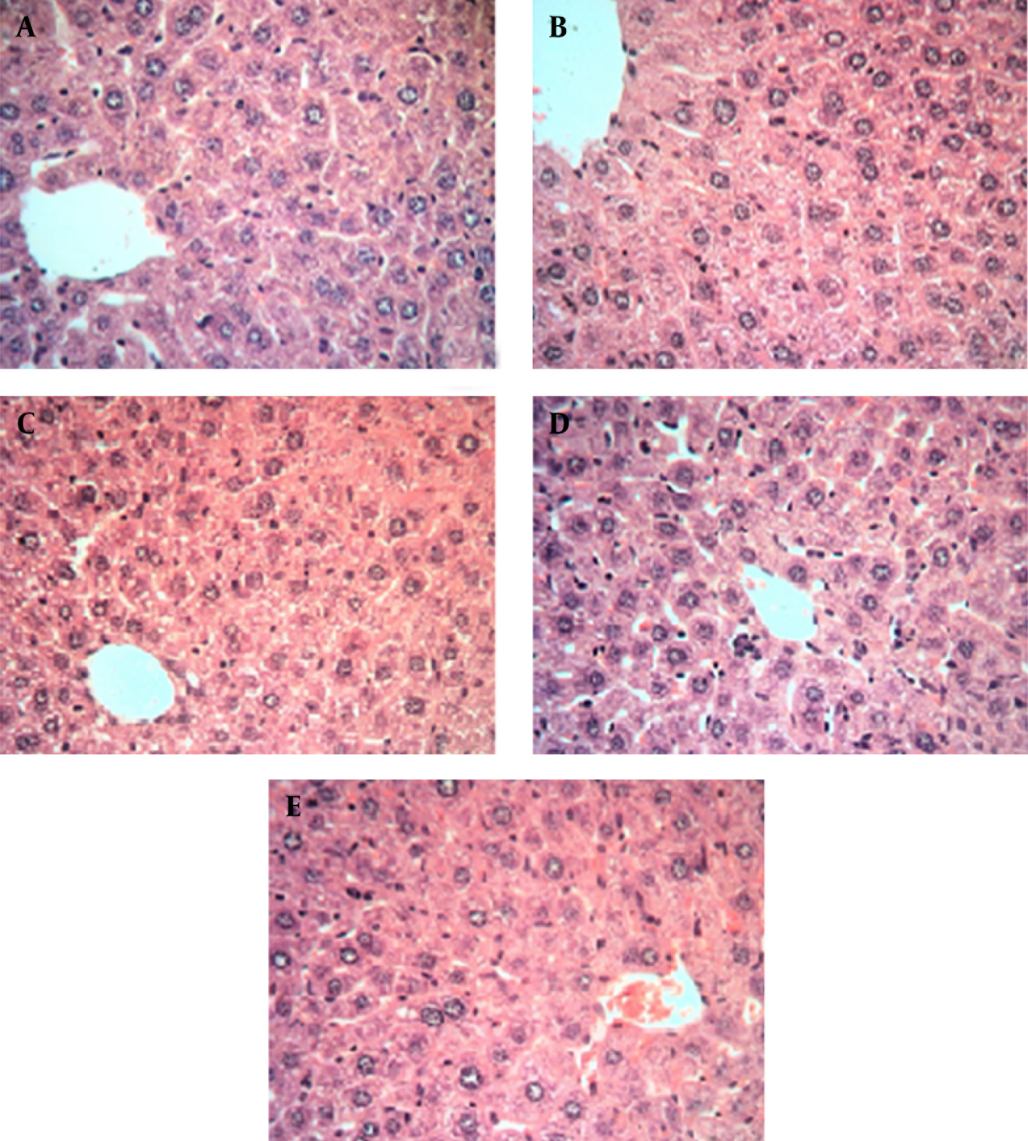
Hepatic Histopathological Evaluation of Albino Mice Treated With Sour Cherry Kernel Extract Microemulsion for 10 Days * Histological staining with hematoxylin and eosin (H&E) at 40X magnification shows the normal liver structure between all groups, showing normal hepatic parenchyma and hepatocytes. A. control group, B. microemulsion base treated group, C. 2.5% treated group, D. 5% treated group, E. 10% treated group.

Histopathological evaluation is an essential aspect of safety assessments, and has supplied supportive evidence for serum biochemical analysis. Results of histopathological examinations of liver samples, stained with hematoxylin and eosin (H&E), are shown in microphotographs (Figure 2 at 40X magnification). It is well known that liver damage can be characterized by structural study of hepatocytes, hepatic cord, and sinusoids. As indicated, in Figure 2 A, mice received only normal saline via oral gavage, in Figure 2 B mice received base microemulsion phase, and the tested groups received 2.5%, 5%, and 10% sour cherry kernel microemulsion extract in Figures 2 C, 2D, and 2E.

Histological profiles of liver and kidney samples of mice at the 10th day of consumption of herbal extract microemulsion with different dosages of microemulsion extract in comprise to normal saline and control group did not show any significant differences. The dosage groups (groups 3, 4, and 5) showed normal parenchymal structures with cords of hepatocytes, portal tracts, and central veins, lacking considerable changes, compared to the normal control group. This result was confirmed about the dosage groups in kidney and its structure properties.

**Figure 3.**
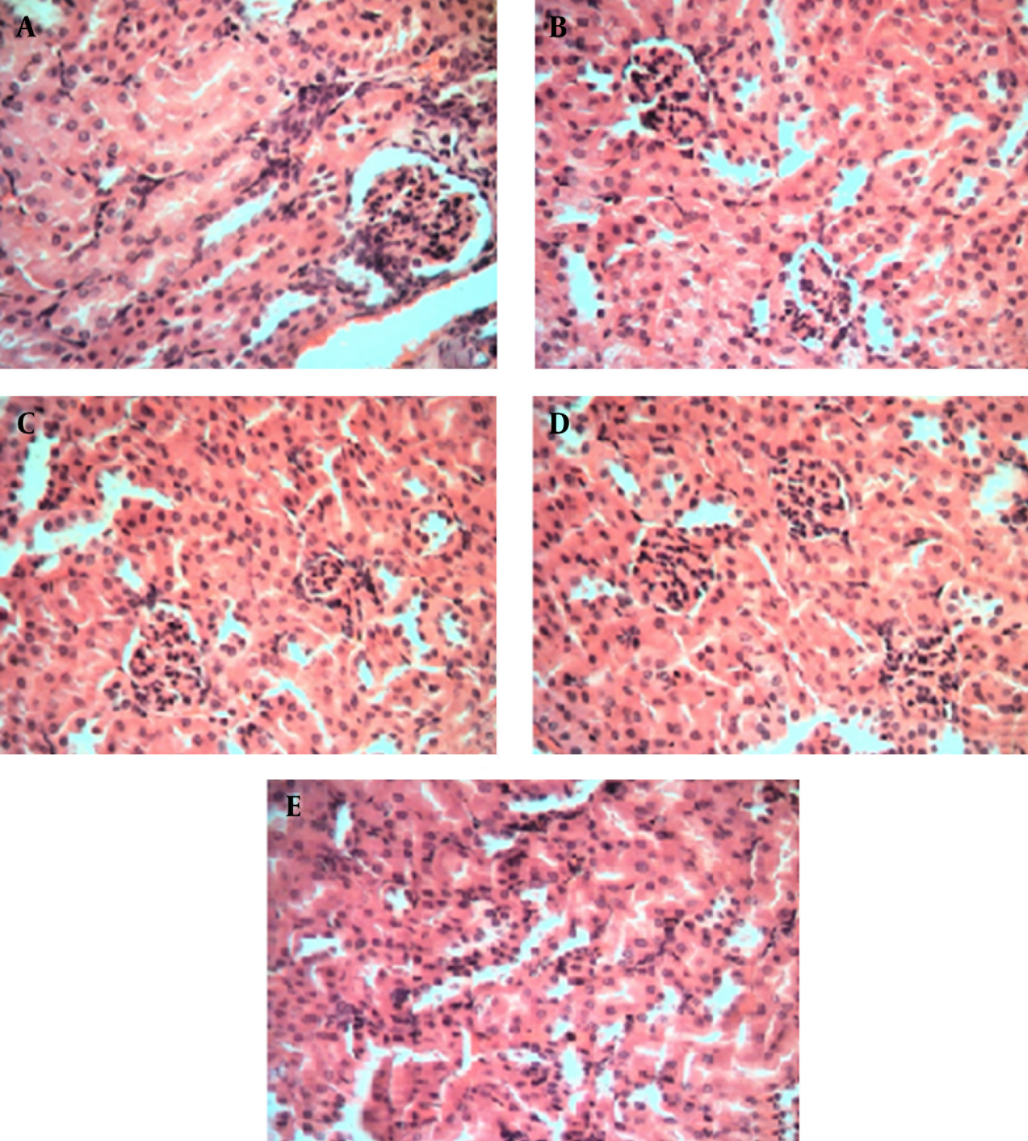
Kidney Histopathological Evaluation of Albino Mice Treated With Sour Cherry Kernel Extract Microemulsion for 10 Days * Histological staining with hematoxylin and eosin (H&E) at 40X magnification shows the normal kidney structure between all groups, there is no significant damage to the glomeruli such as (hypertrophy, hypercellularity) and tubules (dilatation, atrophy). A. control group, B. microemulsion base treated group, C. 2.5% treated group. D. 5% treated group. E. 10% treated group.

Results of histopathological examinations of kidney samples stained with hematoxylin-eosin are shown in Figure 3 at 40X magnification. Kidney damage can be characterized via structural study by ultrastructure finding. As indicated, in Figure 3 A mice received only normal saline via oral gavage, in Figure 3 B mice received base microemulsion phase, and the tested groups received 2.5%, 5%, and 10% sour cherry kernel microemulsion extract in Figures 3 C, 3D, and 3E.

## 5. Discussion

In the present study, mice were used for toxicity evaluation of microemulsion extracts, because mice and humans’ genes are nearly 99 percent similar, and their genome is easily manipulated for different researches through various genetic engineering technologies. Using mice decreases the costs of experiment, their total life stages can be studied, their gestation time is short (proximally 3 weeks), and they can be easily handled (32). Therefore, mice are ideal model organisms for different studies. Sour cherry has been reported to contain individual physiological and pharmacological properties. Most beneficial effects of sour cherry kernel are associated with the presence of antioxidant compounds such as polyphenols, flavonoids, proanthocyanidin and anthocyanidin, trans-resveratrol, and catechins (15). Dosage of the plant extract was computed according to an approved study where the therapeutic dosage to mice was normalized (25). In the present study, toxicity evaluation of microemulsion (nano size) of sour cherry kernel extract for the oral bioavailability enhancement in mice was conducted to determine whether this form of extract (microemulsion) has the same effect as usual form of the sour cherry kernel extract on systemic and dermal toxicity (25), as well as protective effects (14). These can be further fields of researches. In this study, we intended to provide evidence for the safety of extract microemulsion, which was free of toxic effects. These can be further fields of researches. In this study, we intended to provide evidence for the safety of extract microemulsion, which was free of toxic effects. The changes occurred in weights of mice by normal saline, microemulsion phase, and extracts microemulsions, revealed no significant differences.

Of toxicology, Liver and kidneys are major tissues prone to interact with toxic material, thus chiefly responsible for foreign risky materials. These changes include an organ weight loss due to tissue cell death, or an organ weight gain due to edema or abnormal cell proliferation (33). Measuring the blood levels of biochemical enzymes is important to indicate any damage to the tissues. The raised serum levels of ALT, AST, and ALP were ascribed to the injured healthy structure of the liver tissue, because they are cytoplasmic in their position, and are released into blood circulation after cellular injury. High levels of each factor may imply a failure of active pathologies (34, 35). The insignificant effects of microemulsion phase and extracts microemulsions on ALT, AST, ALP, BUN, and creatinine levels after 10 days of in-vivo testing (demonstrated in Table 1), showed that the extracts microemulsions may not be toxic to major key enzymes included in basic metabolic activities of the liver and kidneys. Using the data presented in this report and considering no evidence of toxicity effects of extract microemulsion, we can conclude the bioavailability enhancement of the health benefits of bioflavonoids in sour cherry seed kernel extract and the targeted drug delivery. We observed a better efficacy of sour cherry seed kernel extract as well as its protection against enzymatic hydrolysis and control of their release, its long shelf life, high diffusion and absorption rates, and development of oral bioavailability for active pharmaceutical ingredients useful in protective mechanisms.

## References

[1] Sarciaux JM, Acar L, Sado PA (1995). Using microemulsion formulations for oral drug delivery of therapeutic peptides.. Int J Pharm..

[2] Gao ZG, Choi HG, Shin HJ, Park KM, Lim SJ, Hwang KJ (1998). Physicochemical characterization and evaluation of a microemulsion system for oral delivery of cyclosporin A.. Int J Pharm..

[3] Constantinides PP (1995). Lipid microemulsions for improving drug dissolution and oral absorption: physical and biopharmaceutical aspects.. Pharm Res..

[4] Lin H, Gebhardt M, Bian S, Kwon KA, Shim CK, Chung SJ (2007). Enhancing effect of surfactants on fexofenadine.HCl transport across the human nasal epithelial cell monolayer.. Int J Pharm..

[5] Salimi A, Moghimipour E, Sharif makhmalzadeh B (2013). Preparation and Characterization of Cyanocobalamin (Vit B12) Microemulsion Properties and Structure for Topical and Transdermal Application.. Iran J Basic Med Sci..

[6] Yi DK, Lee SS, Ying JY (2006). Synthesis and applications of magnetic nanocomposite catalysts.. Chem Mater..

[7] Jadhav KR, Shaikh IM, Ambade KW, Kadam VJ (2006). Applications of microemulsion based drug delivery system.. Curr Drug Deliv..

[8] Yin YM, Cui FD, Mu CF, Choi MK, Kim JS, Chung SJ (2009). Docetaxel microemulsion for enhanced oral bioavailability: preparation and in vitro and in vivo evaluation.. J Control Release..

[9] Holm R, Porter CJH, Edwards GA, Müllertz A, Kristensen HG, Charman WN (2003). Examination of oral absorption and lymphatic transport of halofantrine in a triple-cannulated canine model after administration in self-microemulsifying drug delivery systems (SMEDDS) containing structured triglycerides.. Eur J Pharm Sci..

[10] Pouton CW (2000). Lipid formulations for oral administration of drugs: non-emulsifying, self-emulsifying and ‘self-microemulsifying’ drug delivery systems.. Eur J Pharm Sci..

[11] Marshall PJ, Kulmacz RJ, Lands WE (1987). Constraints on prostaglandin biosynthesis in tissues.. J Biol Chem..

[12] Nakamura Y, Matsumoto H, Todoki K (2002). Endothelium-Dependent Vasorelaxation Induced by Black Currant Concentrate in Rat Thoracic Aorta.. Jpn J Pharmacol..

[13] Mazzari S, Canella R, Petrelli L, Marcolongo G, Leon A (1996). N-(2-Hydroxyethyl)hexadecanamide is orally active in reducing edema formation and inflammatory hyperalgesia by down-modulating mast cell activation.. Eur J Pharmacol..

[14] Bak I, Lekli I, Juhasz B, Nagy N, Varga E, Varadi J (2006). Cardioprotective mechanisms of Prunus cerasus (sour cherry) seed extract against ischemia-reperfusion-induced damage in isolated rat hearts.. Am J Physiol Heart Circ Physiol..

[15] Szabo ME, Gallyas E, Bak I, Rakotovao A, Boucher F, de Leiris J (2004). Heme oxygenase-1-related carbon monoxide and flavonoids in ischemic/reperfused rat retina.. Invest Ophthalmol Vis Sci..

[16] Ingi T, Cheng J, Ronnett GV (1996). Carbon Monoxide: An Endogenous Modulator of the Nitric Oxide Cyclic GMP Signaling System.. Neuron..

[17] Stone JR, Marletta MA (1994). Soluble guanylate cyclase from bovine lung: activation with nitric oxide and carbon monoxide and spectral characterization of the ferrous and ferric states.. Biochem..

[18] Matsumoto H, Ishikawa K, Itabe H, Maruyama Y (2006). Carbon monoxide and bilirubin from heme oxygenase-1 suppresses reactive oxygen species generation and plasminogen activator inhibitor-1 induction.. Mol Cell Biochem..

[19] Jiang F, Roberts SJ, Datla S, Dusting GJ (2006). NO modulates NADPH oxidase function via heme oxygenase-1 in human endothelial cells.. Hypertension..

[20] Panahian N, Yoshiura M, Maines MD (1999). Overexpression of heme oxygenase-1 is neuroprotective in a model of permanent middle cerebral artery occlusion in transgenic mice.. J Neurochem..

[21] Suchard JR, Wallace KL, Gerkin RD (1998). Acute Cyanide Toxicity Caused by Apricot Kernel Ingestion.. Ann Emerg Med..

[22] Vough RL, Cassel EK (2000.). Prussic acid poisoning of livestock: causes and prevention..

[23] Schejter A, Ryan MD, Blizzard ER, Zhang C, Margoliash E, Feinberg BA (2006). The redox couple of the cytochrome c cyanide complex: the contribution of heme iron ligation to the structural stability, chemical reactivity, and physiological behavior of horse cytochrome c.. Protein Sci..

[24] Galati G, O'Brien PJ (2004). Potential toxicity of flavonoids and other dietary phenolics: significance for their chemopreventive and anticancer properties.. Free Radic Biol Med..

[25] Bak I, Czompa A, Csepanyi E, Juhasz B, Kalantari H, Najm K (2011). Evaluation of systemic and dermal toxicity and dermal photoprotection by sour cherry kernels.. Phytother Res..

[26] Lapasin R, Grassi M, Coceani N (2001). Effects of polymer addition on the rheology of o/w microemulsions.. Rheologica acta..

[27] Gabbott P (2008). Principles and applications of thermal analysis..

[28] Reitman S, Frankel S (1957). Determination of serum glutamic oxaloacetic transaminase and pyruvic transaminase by colorimetric method.. Am J Clin Path..

[29] King J, King J (1965). The phosphohydrolases-acid and alkaline phosphatases.. Practical clinical enzymology..

[30] Bretaudiere JP, Phung HT, Bailly M (1976). Direct enzymatic determination of urea in plasma and urine with a centrifugal analyzer.. Clin Chem..

[31] Spencer K (1986). Analytical reviews in clinical biochemistry: the estimation of creatinine.. Ann Clin Biochem..

[32] Hedrich H, Hans H (2004). The laboratory mouse..

[33] Voss KA, Riley RT, Norred WP, Bacon CW, Meredith FI, Howard PC (2001). An overview of rodent toxicities: liver and kidney effects of fumonisins and Fusarium moniliforme.. Environ Health Perspect..

[34] Atasoy N, Erdogan A, Yalug I, Ozturk U, Konuk N, Atik L (2007). A review of liver function tests during treatment with atypical antipsychotic drugs: a chart review study.. Prog Neuropsychopharmacol Biol Psychiatry..

[35] Fujii T (1997). Toxicological correlation between changes in blood biochemical parameters and liver histopathological findings.. J Toxicol Sci..

